# Phoenix Dan Cong Tea: An Oolong Tea variety with promising antioxidant and *in vitro* anticancer activity

**DOI:** 10.29219/fnr.v62.1500

**Published:** 2018-11-13

**Authors:** Xiaobin Zhang, Zhenhuan Song, Yuanyuan You, Xiaoling Li, Tianfeng Chen

**Affiliations:** 1 The First Affiliated Hospital, and Department of Chemistry, Jinan University, Guangzhou 510632, China; 2 Institute of Food Safety and Nutrition, Jinan University, Guangzhou 510632, China

**Keywords:** Dan Cong tea, aqueous extract, cell apoptosis, protective effect, free radical scavenging, oxidative damage

## Abstract

**Background:**

Phoenix Dan Cong tea is an Oolong tea produced in Chaozhou, China. Nowaday, the experimental studies on the benefical effects of the Phoenix Dan Cong tea are rare.

**Objective:**

The objective of this study was to comprehensively evaluate the activity of Phoenix Dan Cong tea aqueous extract (PDCe).

**Methods:**

We used a series of evaluation methods in the present study to achieve an in-depth understanding and evaluation of the antioxidant and antitumor activity of PDCe.

**Results:**

High-performance liquid chromatography (HPLC) studies have indicated that PDCe is rich in catechins such as gallocatechin (GC), epigallocatechin (EGCG) and epicatechin gallate (ECG), with sparse amounts of theaflavins. We discovered that PDCe scavenges ABTS•+ and DPPH• free radicals in a dose-dependent manner. In addition, PDCe can significantly induce apoptosis of MDA-MB231 cells, mainly through the death-receptor-mediated extrinsic apoptotic pathway. Internalized PDCe can not only downregulate intracellular reactive oxygen species levels but also induce oxidative damage to mitochondria in MDA-MB231 cells.

**Conclusions:**

Phoenix Dan Cong tea may act as a substitute for natural antioxidants and as a promising anticancer agent due to its protective effect on human health.

Various degenerative and chronic diseases are frequently attributed to oxidative stress, which is often caused by free radicals (1). Free radicals can easily react with the cellular molecules because of their highly reactive and unstable nature, and can oxidize nucleic acids, proteins, and fats, thus promoting degenerative diseases (2). Essential biochemical reactions in the human body as well as external exposure may generate free radicals (3). In general, antioxidants can react with free radicals producing relatively stable substances. The human body does not need to replenish antioxidants under normal circumstances because it continues to synthesize and secrete endogenous antioxidants. However, exogenous antioxidants are needed when free radicals are produced in large quantities (4). It is necessary to maintain a balance between free radicals and antioxidants for normal physiological function of the human body. Natural antioxidants can not only protect the human body from damage caused by reactive oxygen species (ROS) but also inhibit lipid peroxidase activity and thus prevent the degenerative diseases (5). Most importantly, natural antioxidants, such as tea polyphenols, have low toxicity and cause no harm to humans even if used chronically.

Tea has been consumed for thousands of years as a daily health drink in China. Tea has been traditionally used as a medication based on experience, and biological activities including antioxidant and antitumor activities of the active ingredients of tea have been extensively described in China and Japan. The major ingredients in the extract of tea, flavonols and polyphenols, have been proven to be beneficial to the human body (6–8). Active components playing crucial roles in most of the biological activities of tea are known to be catechins (also known as polyphenols) (9, 10). Tea polyphenols are considered responsible for antimutagenic and anticarcinogenic activity, and protection against cardiovascular diseases (11). Teas are mainly classified into green tea (unfermented), Oolong tea (semi-fermented), and black tea (fully fermented) depending on the degree of fermentation in manufacture, where the term fermentation refers to the natural browning reactions resulting from oxidative enzymes in the cells of tea leaves (12). Oolong tea has been the most favored choice among Taiwanese over the past few decades owing to its special taste and flavor (13, 14). Phoenix Dan Cong tea, a variety of Oolong tea, is one of the six tea categories in China. It has a long history and reputation of more than 900 years. The Chaozhou Phoenix mountains are one of the three major Oolong tea producing areas in China and the ‘Phoenix narcissus variety’ of tea owes its origin to these mountains. The Phoenix Dan Cong tea used in the present study has been screened out from the Phoenix narcissus variety by generations of tea farmers. It is one of the most tasty and fragrant varieties of tea in China. Phoenix Dan Cong tea has many health benefits, for example, vitamin C is known to play an important role in skin growth but easily reacts with ROS in the human body; however, the antioxidant effect of tea polyphenols in Phoenix Dan Cong tea is able to eliminate ROS and inhibit the elimination of vitamin C, protecting the skin and enabling its whitening (15–17).

Based on the fact that different varieties of tea are used as health care products worldwide, but experimental studies on the benefical effects of teas such as the Phoenix Dan Cong tea are rare, the present investigation was aimed to comprehensively evaluate the activity of Phoenix Dan Cong tea aqueous extract (PDCe), assessing ABTS•+ and DPPH• levels and antitumor activity using the human tumor cell lines MDA-MB231 (human breast cancer cells) and SW480 (human colon cancer cells). The possible mechanisms involved that were studied were induction of cell cycle arrest and apoptotis.

The MDA-MB231 human breast cancer cell line and SW480 human colon cancer cells were selected as cellular models to evaluate *in vitro* antitumor effects of PDCe, while ABTS•+ and DPPH• free radicals were used to evaluate its antioxidant effects. As shown in Fig. 1A, PDCe not only had an excellent anticancer effect against MDA-MB231 cells but also protected against damage caused by ROS. Our results provide evidence that PDCe may act as a substitute for natural antioxidants and as a promising anticancer agent.

**Fig. 1:**
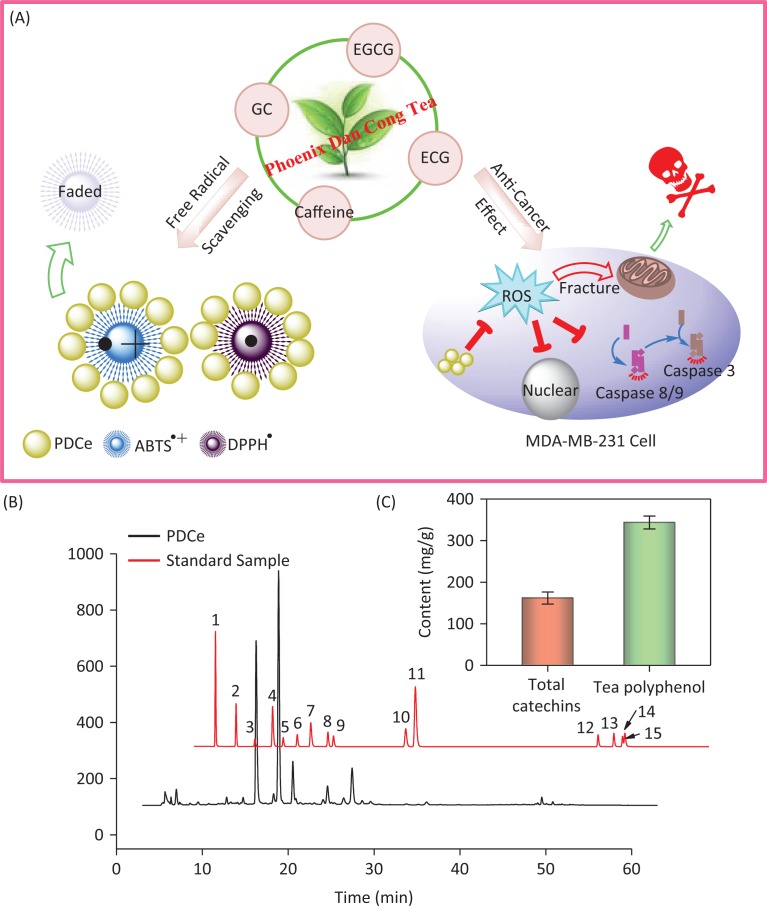
The antioxidant and anti-breast cancer behavior *in vitro* and HPLC analysis of the active ingredients in Phoenix Dan Cong Tea. (A) The anticancer mechanism and the free radical scavenging behavior of PDCe. (B) Representative HPLC-UV chromatogram acquired at 278 nm of 15 standard samples and PDCe. (C) The total catechins and total phenolic contents (TPC) (as GAE) in PDCe. 1: A; 2: GA; 3: GC; 4: theophylline; 5: EGC; 6: C; 7: Caffeine; 8: EC; 9: EGCG; 10: ECG; 11: CG; 12: TF1; 13: TF2; 14: TF3; 15: TF4.

## Materials and methods

### Materials and reagents

Reference standards for caffeine, EC, ECG, CG, C, CA, EGC, and EGCG (≥98%) and those for TF1, TF2, TF3, and TF4 (≥90%) were purchased from Chen du purify Co. (Chen du, China). Folin-Ciocalteu’s phenol reagent was purchased from Sigma (St. Louis, MO). Methanol (HPLC grade), 85% phosphoric acid, acetonitrile (HPLC grade), and Milli-Q water were filtered through a 0.45 μm membrane before use. 6-hydroxy-2,5,7,8-tetramethylchromane-2-carboxylic acid (Trolox), 2,2’-azinobis-3-ethylbenzothiazolin-6-sulfonic acid (ABTS•+), 1,1-diphenyl-2-picryhydrazyl(DPPH•), propidium iodide (PI), thiazolyl blue tetrazolium bromide (MTT), glutathione (GSH), 4’ 6-diamidino-2-phenylindole (DAPI), bicinchoninic acid (BCA), sodium selenite, and all other chemicals were obtained from Sigma-Aldrich (St. Louis, MO, USA). Fetal bovine serum (FBS) and antibiotic mixture (penicillin–streptomycin) were purchased from Invitrogen (Carlsbad, CA, USA). Caspase-3, caspase-8, and caspase-9 were purchased from Cell Signaling Technology (Beverly, MA, USA). Caspase-3, caspase-8, and caspase-9 substrates were obtained from Biomol (Germany).

### Preparation of PDCe

A dry fine powder of Phoenix Dan Cong tea was purchased from the local tea processing plant. The powder was stored at 4 C, and when necessary, it was dissolved in phosphate-buffered saline (PBS) to make a 10 mg/mL working solution.

### Measurement of total polyphenol content

Total polyphenol content (TPC) was measured using spectrophotometric detection of gallic acid (GA) per the Folin–Ciocalteu method (18, 19). Each sample was measured in triplicate under the same conditions. The procedures were repeated for standard solutions. The absorbance of the mixture was measured at 765 nm with water as blank using a spectrophotometer (Genesys5, Spectronic Instruments, Rochester, NY). The TPC was expressed as gallic acid equivalents (GAE) in milligram of GA pergram of tea extract.

### HPLC analysis of catechins and theaflavins in PDCe

High-performance liquid chromatography (HPLC) (20–22) was used to measure the catechin and theaflavin content of PDCe. HPLC was performed using a 1260 infinity II chromatography system from Agilent Technologies. PDCe was injected onto a SiO_2_ column (250×4.6 mm), previously equilibrated with a solution composed of solvent A (acetonitrile) and solvent B (0.4% aqueous phosphoric acid, v/v). Compounds were eluted from the column using the following program: 7–15% A in 0–13 min, 15–20% A in 13–35 min, 20–50% A in 35–70 min, 50–80% A in 70–90 min, 80–87% A in 90–115 min. The flow-rate of the chromatographic mobile phase was set as 1.0 mL/min and the effluent was detected at 278 nm for acquiring chromatograms.

### ABTS•+ scavenging assay

The ABTS•+ scavenging assay as previously described (23) was applied to evaluate the antioxidant activity of PDCe. The absorbance of the solution was measured at 734 nm after the initiation of mixing for 1 min. The antioxidant capacity of PDCe was evaluated by calculating half maximal inhibitory concentration (IC_50_). The scavenging assay was performed using a spectrophotometer (Genesys 5, Spintronic Instruments, Rochester, NY).

### DPPH• scavenging assay

The DPPH• scavenging activity of PDCe was evaluated using a spectrophotometer (Genesys5, Spectronic Instruments, Rochester, NY) following the method described by Chen and Wong (2008a) (23). The change in absorbance of a mixture that was left to stand for 5 min at 515 nm was measured. Half maximal inhibitory concentration (IC_50_) was calculated to evaluate the antioxidant capacity of PDCe.

### Cell culture

Human cell lines used in this study included HeLa cervical cancer cells, SW480 human colon cancer cells, MDA-MB231 human breast cancer cells, HepG2 hepatocellular carcinoma cells, WI38 human lung cells, and L02 human normal liver cells, and they were purchased from the American Type Culture Collection (ATCC, Manassas, Virginia). The cell lines were cultured in DMEM media, with penicillin (100 units/mL), streptomycin (50 units/mL), and FBS (10%) at 37°C in a humidified incubator under 5% CO_2_.

### Cell viability assay

Changes in cell viability induced by the PDCe was determined using MTT assay, based on a previous study (24). In short, the cell viability (2 × 10^4^ cells per mL for cancer cells and 4× 10^4^ cells per mL for normal cells) after treatment with different concentrations of PDCe for 72 h was determined using an MTT assay. A micro-plate spectrophotometer (Spectro Amax TM 250) was used to measure the color intensity of the formazan solution at 570 nm which reflected the growth of the cells (25).

### Flow cytometric analysis

Cell cycle distribution was analyzed via flow cytometry as previously described (26, 27). In short, MDA-MB-231 cells treated with PDCe were washed with PBS and then treated with 5% trypsin, then fixed in 75% ethanol overnight at −20°C. Subsequently, the fixed cells were stained with PI in darkness. The stained cells were analyzed using an FC-500 flow cytometer (Beckman Coulter, Miami, FL). Cell cycle distribution was analyzed using the software Multi-Cycle (Phoenix Flow Systems, San Diego, CA). The proportion of cells in G0/G1, S, and G2/M phases was represented in the DNA histogram. Apoptotic cells with hypodiploid DNA content were measured by quantifying the sub-G1 peak in the cell cycle pattern. A total of 10,000 events were recorded in each experimental sample.

### Caspase activity assay

Caspase activity was determined via fluorescence intensity measurement using specific caspase-3, caspase-8, and caspase-9 substrates as reported (28). Specifically, harvested cells pellets were suspended in cell lysis buffer (Beyotime) and incubated on ice for 1 h. After centrifugation at 11,000 g for 30 min, the BCA assay was immediately performed to measure protein concentration in the supernatants. Thereafter, the cell lysates and specific caspases substrates (Ac-DEVD-AMC for caspase-3, Ac-IETD-AMC for caspase-8, and Ac-LEHD-AMC for caspase-9) were mixed at specific ratios in 96-well plates and incubated at 37°C for 2 h. The fluorescence intensity of the mixtures which reflected the caspase activity was detected at excitation and emission wavelengths of 380 nm and 460 nm, respectively.

### Measurement of intracellular ROS generation

The relative levels of ROS were determined using fluorometric assays (DHE and DCFH-DA assay) (29, 30). The generation of ROS was determined via fluorescence intensity measurement using a multifunction spectrometer (Bio-Tek^®^, ELX 800, American) at excitation and emission wavelengths of 300 nm and 600 nm, respectively. Relative DHE and DCFH-DA fluorescence intensity of the treated cells was expressed as a percentage of control (as 100%).

### Mitochondrial fragmentation analysis

Mitochondrial fragmentation analysis was carried out as reported (31). Briefly, mitochondria and nuclei of the MDA-MB231 cells were stained with Mito Tracker Red CMXRos and H33342, respectively. Prior to that, the cells were treated with PDCe (30 μg/mL) for 0, 6, or 12 h, and following straining, photographed using a monochromatic Cool SNAPFX camera (Roper Scientific, USA).

### Statistical analysis

Results were expressed as mean ± SD, which were obtained from at least three independent experimental results. The difference between the two groups was analyzed using a two-tailed Student’s *t*-test. Differences with *P < 0.05* (*) or *P < 0.01* (**) were considered statistically significant. One-way analysis of variance (ANOVA) was used to compare multiple groups.

## Results and discussion

### Measurement of the active ingredients in PDCe

Because several tea extracts are now available, the concentrations of components are known and standard solutions with suitable concentration ranges are available for analysis; these would reduce error and enable reproducibility and credibility of the results. Therefore, the concentrations of different components in the extract was calculated using a calibration curve method. Fig. 1B shows the typical HPLC-UV chromatogram at 278 nm of the PDCe and standard samples mixture including catechins and theaflavins. The retention times and spectra were compared with those of commercially available catechins and theaflavins. The peaks and retention times of PDCe components were compared to those of the standard compounds, and 15 active compounds were identified in the PDCe (Table 1). Compounds such as GC, GA, and ECG, and especially EGCG and caffeine, were abundant, while ingredients such as EC, theophylline, and CG were low. It is worth mentioning that there were almost no theaflavins in the PDCe. In addition, as shown in Fig. 1C, the TPC of PDCe was 343.6 mg/g and the total catechin content was 161.9 mg/g, which accounted for approximately 47.1% of the TPC. Taken together, our data allow us to conclude that GC, EGCG, ECG, and caffeine are the major catechins in PDCe, while theaflavins are rare. These tea polyphenols or caffeine in the PDCe may play an important role in its antioxidant activity and *in vitro* antitumor activity.

**Table 1:** Chemical composition analysis of PDCe by HPLC analysis

Peak	Retention Time (min)	Compound	Contents (%)
1	2.436	A	0.31
2	4.375	GA	1.55
3	6.467	GC	3.89
4	8.771	Theophylline	0.04
5	9.890	EGC	0.82
6	11.56	C	0.80
7	13.36	Caffeine	5.21
8	15.34	EC	0.76
9	15.97	EGCG	6.15
10	24.52	ECG	1.58
11	25.66	CG	0.63
12	47.14	TF1	0.09
13	48.97	TF2	0.21
14	49.98	TF3	0.06
15	50.24	TF4	0.06

### Determination of the optimum wavelength

**Fig. 2:**
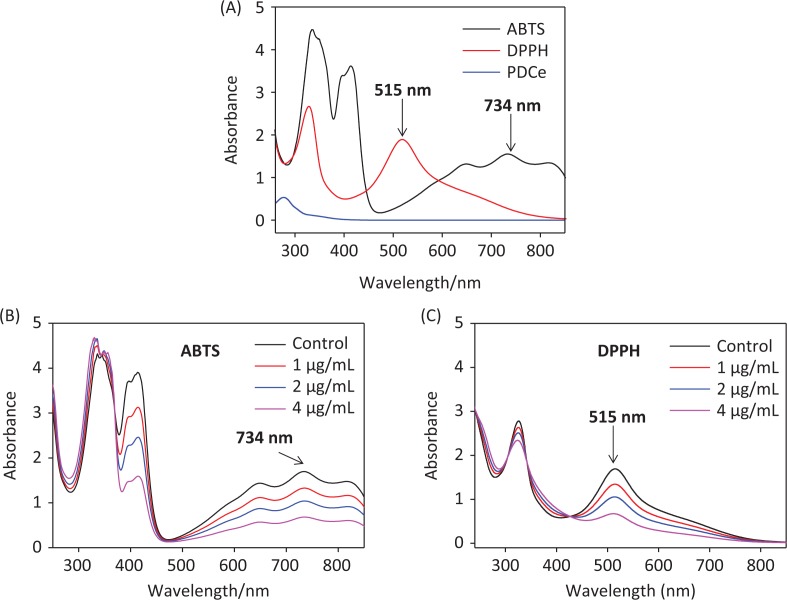
Ultraviolet absorbance spectra. (A) Absorbance spectra of PDCe and the ABTS•+ and DPPH• solutions. (B) Changes in absorbance spectra of ABTS•+ solution with the addition of PDCe. (C) Changes in absorbance spectra of DPPH solution with the addition of PDCe.

ABTS•+ and DPPH• free radical scavenging has been widely used in the measurement of total antioxidant capacity of test samples (25). In the test, the ABTS•+ and DPPH• solution were subjected to UV-Vis spectral scanning. As shown in Fig. 2A, the ABTS•+ solution showed characteristic absorption peaks at 734 nm and 415 nm, while the characteristic absorption peak of the DPPH• solution was at 515 nm. We discovered that the PDCe had no UV absorption at 515 nm or 734 nm, but there was slight ultraviolet UV absorption at 415 nm. Further, the absorption peak at 734 nm (ABTS•+) and 515 nm (DPPH•) showed dose-dependent inhibition and excellent linearity correlation after the addition of PDCe (Fig. 2B and C). The results above indicated that the change in absorption peaks at 734 nm and 515 nm reflect scavenging of ABTS•+ and DPPH free radicals by PDCe to some extent. Therefore, we selected 734 nm and 515 nm as the detection wavelengths in the two free radical scavenging experiments, the ABTS and DPPH assays, respectively.

### Antioxidant activity evaluation

**Fig. 3:**
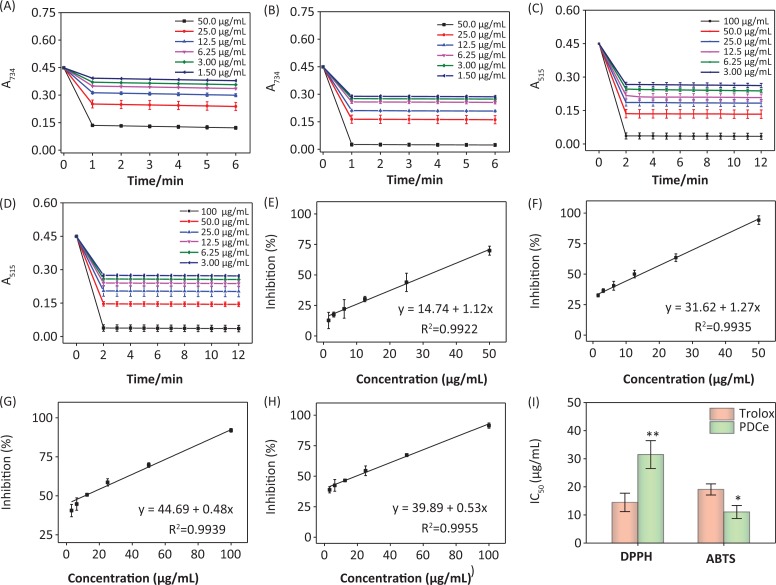
Effect of PDCe and Trolox in free radicals scavenging. (A) Changes in absorbance spectra of ABTS•+ solution with the addition of PDCe. (B) Changes in absorbance spectra of ABTS•+ solution with the addition of Trolox. (C) Changes in absorbance spectra of DPPH• solution with the addition of PDCe. (D) Changes in absorbance spectra of DPPH• solution with the addition of Trolox. (E) Dose-dependent inhibitions of PDCe on ABTS•+ and its linearity correlation. (F) Dose-dependent inhibitions of Trolox on ABTS•+ and its linearity correlation. (G) Dose-dependent inhibitions of PDCe on DPPH• free radicals and its linearity correlation. (H) Dose-dependent inhibitions of PDCe on DPPH• free radicals and its linearity correlation. (I) The inhibitions of the antioxidants on ABTS•+ and DPPH• free radicals. The IC_50_ values of PDCe and the standard antioxidant Trolox determined by ABTS•+ and DPPH• assays. * and ** indicate statistical difference at *P < 0.05* and *P < 0.01*, respectively, by comparing with the Trolox group.

Determination of the response time of the system is known to be a very important factor in evaluating different antioxidants using the ABTS•+ and DPPH• radical scavenging assays. To understand the reaction kinetic characteristics of the ABTS•+ and DPPH• free radical systems, we measured absorbance change kinetics of the ABTS•+ and DPPH• solutions after treating with PDCe or Trolox (as positive control). According to the results of UV spectral scanning shown in Fig. 3A and B, when different concentrations of PDCe and Trolox was added to the ABTS•+ system, the characteristic absorption peaks (A734) of the ABTS•+ system decreased significantly in 60 sec, and tended to be steady after 6 min. As illustrated in Fig. 3C and D, the characteristic absorption peaks (A515) of the DPPH• system decreased significantly in 120 sec and tended to be steady after 12 min following treatment with PDCe or Trolox.

The ABTS and DPPH assays were used in the present study to evaluate the free radical scavenging activity of the PDCe because they can accommodate many experimental samples and also have sufficient sensitivity to detect antioxidant activity at low concentrations. For ABTS•+, the IC50 values of the corresponding antioxidants (PDCe or Trolox) were calculated based on free radical scavenging at the optimum wavelengths (734 nm) and reaction times (60 sec). The same method was applied to DPPH•. In the range of 1.50–50.0 μg/mL, PDCe and Trolox had excellent linear relationships to ABTS•+ scavenging (Fig. 3E and F) and the IC50 values could be calculated; the IC50 values of PDCe and the standard antioxidant Trolox (as positive control) were 11.06 ± 2.32 and 19.08 ± 1.98 μg/mL, respectively (Fig. 3I). In the DPPH• free radical system, PDCe and Trolox also showed good linear relationships to free radical scavenging in the range of 3.00–100 μg/mL (Fig. 3G and H). As shown in Fig. 3I, the IC50 values of PDCe and Trolox in DPPH• scavenging were 31.48 ± 4.96 and 14.47 ± 3.28 μg/mL, respectively; this suggested that PDCe also has an excellent scavenging effect on lipophilic free radicals (DPPH•), although its free radical scavenging ability is not as good as Trolox. Based on the results discussed above, it is clear that PDCe has good antioxidant activity. We can also conclude that PDCe has an excellent inhibitory effect on water-soluble free radicals (ABTS•+) and a lesser effect on lipid soluble free radicals (DPPH•).

### Cytotoxicity of PDCe

**Fig. 4:**
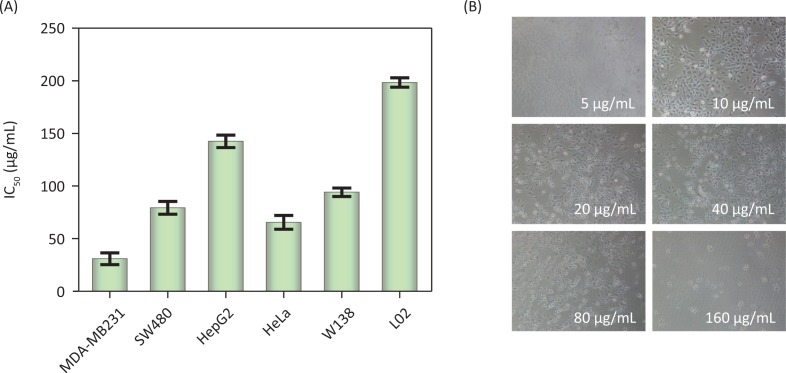
Cytotoxic effects of the PDCe. (A) Antitumor activity of PDCe on various tumor and normal cells. Each value represents means ± SD (n = 3). (B) Effect of the PDCe on the MDA-MB231 breast cancer cells *in vitro*. The cells’ pictures were taken after treating with PDCe for 72 h. Original magnification: 10×.

The anticancer effect of PDCe was evaluated using various human cancer and normal cell lines via MTT assay. The IC50 values obtained in the cytotoxicity assays are shown in Table 2. As shown in Fig. 4A, we found that PDCe had significant cytotoxicity towards MDA-MB-231 cells and SW480 cells with IC50 values of 30.90 ± 5.55 and 79.33 ± 0.06 μg/mL, respectively. However, the PDCe demonstrated relatively low cytotoxicity against L02 and WI38 cells, with IC_50_ values of 198.3 ± 3.48 and 94.1 ± 3.98 μg/mL strongly indicating that PDCe is more cytotoxic to cancer cells (MDA-MB-231, SW480, HeLa) than normal cells (L02 and WI38). It is worth mentioning that the IC50 of PDCe against liver cancer cells (HepG2) was 142.4 ± 2.96 μg/mL, which was higher than that against normal human lung cells (WI38). Further, as shown in Fig. 4B, the PDCe had a dose-dependent effect on MDA-MB-231 cell death. Cell death at PDCe concentrations above 40 μg/mL can be clearly seen. Therefore, MDA-MB231 breast cancer cells and SW480 colon cancer cells, which showed the most sensitivity to PDCe, were selected to investigate the specific mechanisms involved in the anticancer activity of PDCe based on the results of the MTT assay.

**Table 2:** Cytotoxicity Effects of PDCe

Cell Name	IC_50_ (μg/mL)
L02	198.3 ± 4.48
WI38	94.1 ± 3.98
MDA-MB231	30.9 ± 5.55
SW480	79.3 ± 6.06
HepG2	142.4 ± 5.96
HeLa	65.5 ± 6.53

### Flow cytometry analysis of cellular apoptosis or arrest

**Fig. 5:**
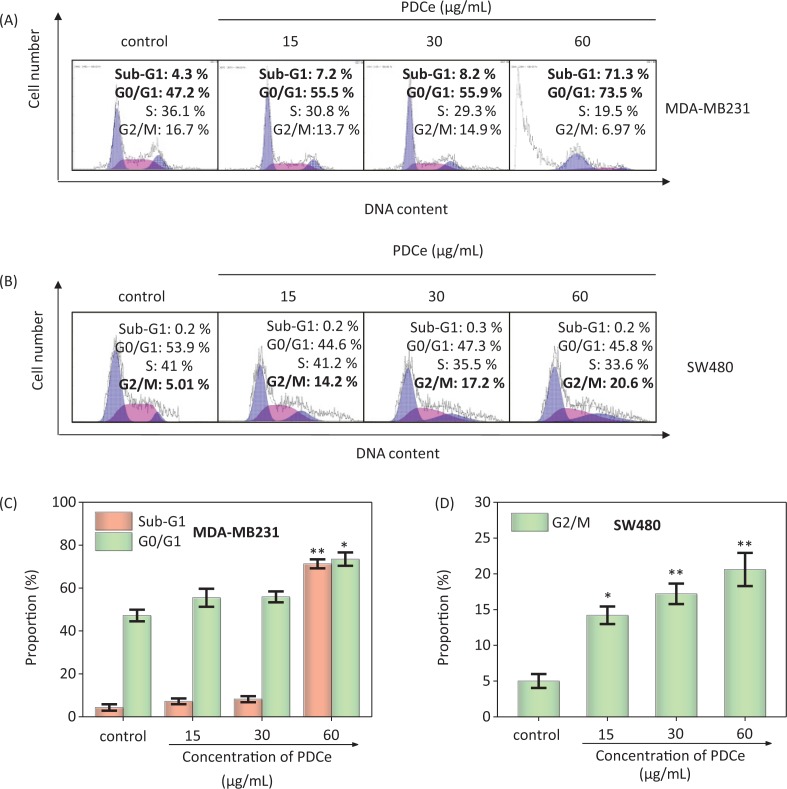
Flow cytometric analysis of MDA-MB231cells and SW480 cells after treatment of PDCe for 72 h. (A) Cell cycle changes of MDA-MB231 cells treated with PDCe for 72 h by flow cytometry analysis. (B) Cell cycle changes of SW480 cells treated with PDCe for 72 h by flow cytometry analysis. (C) Quantitative analysis of sub-G1 and G0/G1 proportion by PDCe in MDA-MB231 cells. (D) Quantitative analysis of G2/M proportion by PDCe in SW480 cells. Each value represents means ± SD (n = 3). * and ** indicate statistical difference at *P < 0.05* and *P < 0.01*, respectively, by comparing with the control group.

Cell cycle arrest and apoptosis are known to be two major routes causing cell death (32–34). In different biological phenomena or systems including cell division, embryonic development, the immune system, chemically induced cell death, and morphological changes, apoptosis is indispensable (35). It has also been shown that cellular apoptosis is an important mechanism in the antitumor activity of natural extracts (36). We performed flow cytometry to analyze the effect of PDCe on the cell cycle distribution of MDA-MB231 breast cancer cells. As shown in Fig. 5A, it is clear that the proportion of sub-G1 peaks of MDA-MB231 cells were significantly dose-dependently increased after PDCe treatment for 72 h, the proportion of sub-G1 peaks was only 8.2% when the concentration of PDCe was 30 μg/mL. However, when the concentration of PDCe was doubled, the proportion of sub-G1 peaks was increased to 71.3%, indicating that more than two-thirds of the MDA-MB231 cells were dead. Moreover, there was a slight dose-dependent increase in the proportion of MDA-MB231 cells in the G0/G1 phases due to PDCe (Fig. 5C). It is worth mentioning that when SW480 cells were treated identically (Fig. 5B), only 0.2% of them were found to be dead at 60 μg/mL PDCe concentration, but the proportion of cells in the G2/M phase showed a significant dose-dependent increase after PDCe treatment (Fig. 5D), which further indicated that the mechanism of PDCe-induced SW480 cell death involved a G2/M phase block rather than apoptosis.

### Caspase activation induced by PDCe

**Fig. 6:**
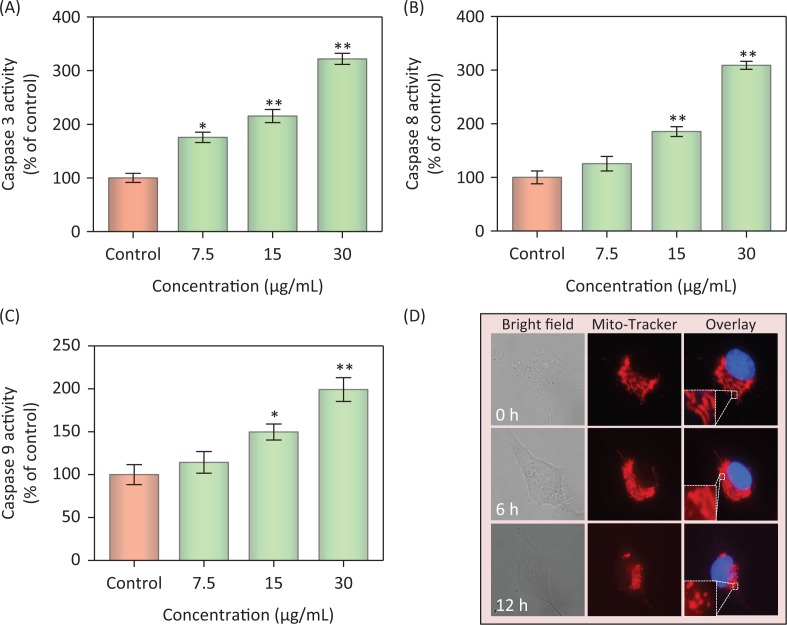
Activation of extrinsic and intrinsic apoptotic pathway by PDCe. (A–C) Quantitative analysis of caspase activation triggered by PDCe. MDA-MB231cells were treated with PDCe for 48 h. Significant difference between treatment and control groups is indicated at * *P < 0.05*, ** *P < 0.01* level. (D) PDCe inhibits mitochondrial fragmentation: representative images of mitochondrial fragmentation in MDA-MB231 cells after treatment with 30 μg/mL PDCe for 12 h. Mitochondria fragmentation was measured by using a fluorescence microscope. Original magnification: 100×.

Caspases, cysteine-containing aspartic acid proteolytic enzymes, are a group of cytoplasmic proteases. Caspases are closely associated with cellular apoptosis (37). Caspase-3 plays a key role in apoptosis; it acts as a central regulator, while caspase-8 and caspase-9 act as the initiators of the exogenous death receptor-mediated and endogenous mitochondria-mediated apoptotic pathways, respectively (38). To assess caspase activity in MDA-MB231 cells during PDCe-induced apoptosis, we measured the fluorescence intensity of substrates of caspase-3, -8, and -9, which indicate the activation of the corresponding enzymes after treatment with PDCe at 7.5, 15, or 30 μg/mL. The results shown in Fig. 6A–C indicate that PDCe could activate caspase-3, caspase-8, as well as caspase-9 in MDA-MB231 cells at different PDCe concentrations, including at the highest concentration of 30 μg/mL. This suggested that both the death receptor-mediated and the mitochondria-mediated pathways are involved in PDCe-induced apoptosis. The activation level of caspase-8 was clearly higher than that of caspase-9 in MDA-MB231 cells after PDCe treatment.

### PDCe-induced intracellular mitochondrial fragmentation

Mitochondria are known to play a key role in cellular activities, but some factors can damage the structure and function of mitochondria and further induce cell apoptosis (39). Fluorescence microscopy-based imaging was used to monitor changes in mitochondrial morphology after PDCe treatment. In the test, two special fluorescence trackers including Mito Tracker (red) and DAPI (blue) were used to label mitochondria and nuclei of cancer cells (Fig. 6D). Initially, the mitochondria were present as red thread-like filaments, and we observed mitochondrial fragmentation and aggregation over time; the morphology was not singnificantly altered at 6 h, but the mitochondria were fragmented into small particles at 12 h after the addition of PDCe. These results suggested that PDCe had a clear impact on mitochondria, specifically causing mitochondrial fragmentation.

### PDCe-induced downregulation of intracellular ROS generation

**Fig. 7:**
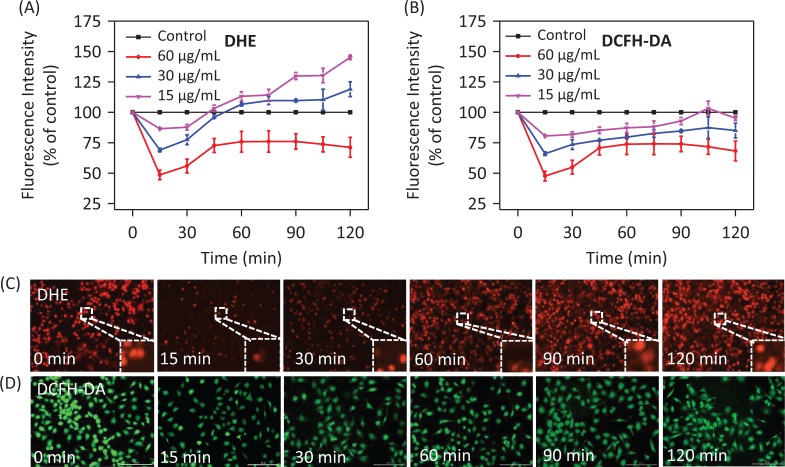
Changes in ROS generation induced by PDCe. (A) The change of the intracellular ROS levels (DHE) in MDA-MB231 cells after being treated with PDCe. All experiments were performed in triplicate. (B) The change of the intracellular ROS levels (DCFH-DA) in MDA-MB231 cells after being treated with PDCe. All experiments were performed in triplicate. Fluorescence imaging of ROS generation in MDA-MB231cells after the incubation of PDCe (30 μg/mL) for indicated times using a DHE (C) and DCFH-DA (D) probe, respectively. Original magnification: 10×.

It has been found that ROS and RNS generation play a key role in the oxidative damage to islet cells. Several DNA components can be subject to attack due to excess ROS in cells, thus causing DNA damage (40, 41). In addition, an intermediate ROS level is a key factor in several cell signaling pathways (42, 43). We assessed intracellular ROS level via measurement of DHE fluorescence intensity after treatment with different concentrations of PDCe. As shown in **Fig. 7A**, ROS generation in MDA-MB231 cells declined rapidly after treatment with PDCe at different concentrations for 15 min, and increased slowly after 15 min showing a significant dose-dependence. To visually verify that PDCe could downregulate ROS, we imaged red fluorescence of DHE in MDA-MB231 cells using microscopy. As shown in Fig. 7C, it is clear that the fluorescence intensity was weakest at 15min, and weaker than that at 0 min at all other times, consistent with the ROS curve shown in Fig. 7A. Further, we found the same phenomenon using the DCFH-DA Test (Fig. 7B and D), with the fluorescence intensity being the weakest at 15 min and subsequently increasing. Taken together, our results show a significant change in ROS generation in MDA-MB231 cells due to the actions of PDCe, which finally induced cell apoptosis. This indicates that PDCe can not only significantly scavenge ABTS•+ and DPPH• free radicals but also cause remarkable clearance of ROS in MDA-MB231 cells.

## Conclusions

In conclusion, the results of the experiments above strongly suggest that PDCe has excellent antioxidant activity, effectively scavenging DPPH• and ABTS•+ free radicals *in vitro*. Further, the inhibition of water-soluble free radicals (ABTS•+) was significantly higher than that of fat-soluble radicals (DPPH•). In addition, PDCe has excellent cytotoxicity against cancer cells, inducing cancer cell death via apoptosis and cell cycle arrest at the G2/M phase. In breast cancer cells, PDCe could not only downregulate intracellular ROS level significantly, causing mitochondrial rupture, but could also induce apoptosis by activating the mitochondria-mediated apoptotic pathway. Overall, our study provides valuable information regarding the benefical effects of Phoenix Dan Cong tea on human health, and that it can act as a natural antioxidant and a promising anticancer agent.

## Funding

This work was supported by Natural Science Foundation of China (21877049), National Program for Support of Top-notch Young Professionals (W02070191), YangFan Innovative & Entepreneurial Research Team Project (201312H05) and Fundamental Research Funds for the Central Universities.
